# Dimensionless Analysis for Investigating the Quality Characteristics of Aluminium Matrix Composites Prepared through Fused Deposition Modelling Assisted Investment Casting

**DOI:** 10.3390/ma12121907

**Published:** 2019-06-13

**Authors:** Sunpreet Singh, Chander Prakash, Parvesh Antil, Rupinder Singh, Grzegorz Królczyk, Catalin I. Pruncu

**Affiliations:** 1 Department of Mechanical Engineering, Lovely Professional University, Phagwara, Punjab 144411, India; snprt.singh@gmail.com (S.S.); chander.mechengg@gmail.com (C.P.); 2 Department of Basic Engineering, College of Agricultural Engineering and Technology, CCS HAU, Hisar, Haryana 125004, India; parveshantil.pec@gmail.com; 3 Manufacturing Research Laboratory, Production Engineering Department, Guru Nanak Dev Engineering College, Ludhiana 141006, India; rupinderkhalsa@gmail.com; 4 Faculty of Mechanical Engineering, Opole University of Technology, 76 Proszkowska St., 45-758 Opole, Poland; g.krolczyk@po.opole.pl; 5 Mechanical Engineering, Imperial College London, Exhibition Rd., London SW7 2AZ, UK; 6 Mechanical Engineering, School of Engineering, University of Birmingham, Birmingham B15 2TT, UK

**Keywords:** fused deposition modelling, investment casting, mathematical modelling, aluminium matrix composite

## Abstract

The aluminium matrix composites (AMCs) have become a tough competitor for various categories of metallic alloys, especially ferrous materials, owing to their tremendous servicing in the diversified application. In this work, additional efforts have been made to formulate a mathematical model, by using dimensionless analysis, able to predict the mechanical characteristics of the AMCs that have already been optimized and characterized by the authors. Here, the experimental and statistical data obtained from the Taguchi L18 orthogonal array and analysis of variance (ANOVA) have been used. They permit collection of the output responses and allow the identification of significant process parameters, respectively, which thereafter were used to design the mathematical model. Second order polynomial equations have been obtained from the specific output response and the relevant input parameter were incorporated with the highest level of contribution. The obtained quadratic equations indicate the regression values (R^2^) equal to unity, hence, proving the performances of the fit. The results demonstrate that the developed mathematical models present very high accuracy for predicting the output responses.

## 1. Introduction

In the last two decades, the rapid advancement of technology has contributed to large modification in the manufacturing sector. During this period, the demand for materials that can sustain the extreme level of service conditions increased globally. Specifically, in aerospace and automobile sectors, the requirement of materials having high strength, toughness, hardness, and prolonged service life was always a challenge. Apart from these properties, one of the major requirements is ‘light weight’. Different studies have reported the needs of lighter material as one of the motivations behind the invention of reinforced materials, commonly, referred to as metal matrix composites [1,2,3]. Amongst various categories of metal matric composites, the one based on the aluminium (Al) matrix is in high demand, owing to its excellent thermal, mechanical, tribological, chemical, and structural characteristics [4,5,6]. Further, there exists a wide range of manufacturing processes, which can be used for the fabrication of the tailor-made composites with desirable properties [7]. Specifically, aluminium matrix composites (AMCs) are basically popular because of the low weight/density ratio, high wear resistance, cost effectiveness, high elastic modulus, and excellent strength [8,9,10,11]. Further, Sajjadi et al. reveal Al-Al_2_O_3_ as the most popular type of AMCs because it contains micro Al_2_O_3_ particles within the matrix of Al [12]. Such as, the Al-Al_2_O_3_ based composites have continually extended their applicability within industrial applications [13,14]. 

Traditionally, the reinforcements are introduced to the metallic matrix via an ex-situ method [15,16], wherein the matrix and reinforcements are mixed with each other outside the mould cavity or die. This method of reinforcement results in poor wettability between the reinforcement and the matrix due to the increased surface area and presence of surface contamination on the reinforcements [17]. In order to overcome the interface issue, recent trends have been shifted towards the use of reinforcements within the cavity or mould itself [18]. As defined in the literature, there are various routes, commercially available, for the preparation of AMC. The most widely used commercial routes are the stir casting and powder metallurgy [19,20,21]. There, the machinability of AMCs is very poor, in contrast to pure Al, due to the brittle reinforcements in the matrix. The investment casting (IC) process has shown its superiority, over the other solutions; in-terms of producing highly complex and near-net shaped parts with very fine surface finish [22]. Additionally, this process is simple, cost effective, and allows manufacturing a wide range of materials; however, the stretched production cycles represents one of the critical challenges for the IC [23,24]. As continuum improvement, the hybridization of IC and stir casting process has become the most popular methods to develop superior metal matrix composites (MMCs) [25,26]. The intrinsic weaknesses of IC process (such as: low strength of wax pattern, un-economical injection moulding cost, high die design cost, and longer production runs) can be eliminated by using fused deposition modelling (FDM) process for pattern making [27,28,29,30]. FDM works on the same principle as the Additive Manufacture (AD), wherein the thin plastic slices are deposited at a defined distance. The interface, therefore, can result in poor surface finish due to an integral stair-casing. Boschetto and Veniali suggested the barrel finishing of formed FDM parts as an efficient method to enhance their surface finish [31]. 

The collaboration of FDM and IC has been extensively researched in the literature, which duly cited the myriads of merits [32,33,34,35,36]. In the recent years, the authors have investigated a novel method for the production of the in-situ based AMCs, through the use of FDM assisted by the IC process. In this respect, the authors developed in-house composite polymeric composites that were used for the production of sacrificial patterns for the IC process. As observed in [1,2,37], the manufactured Al castings consist of Al_2_O_3_ distributions, which permit the validation of the authenticity of the adopted methodology. Further, the input process parameters; refer to Appendix A (i.e., Table A1), have been optimized by using Taguchi L18 orthogonal array-based design of experimentation techniques in response of dimensional accuracy [1], surface hardness [2], and surface roughness [3]. In this work, mathematical models, based on the obtained results of [1,2,3], for all the aforementioned output responses have been developed by using dimensionless modelling, Buckingham’s π-approach. Further, regression equations have been implemented against the best features of the input process parameters, as per analysis of variance (ANOVA).

## 2. Materials and Methods 

**Figure 1:**
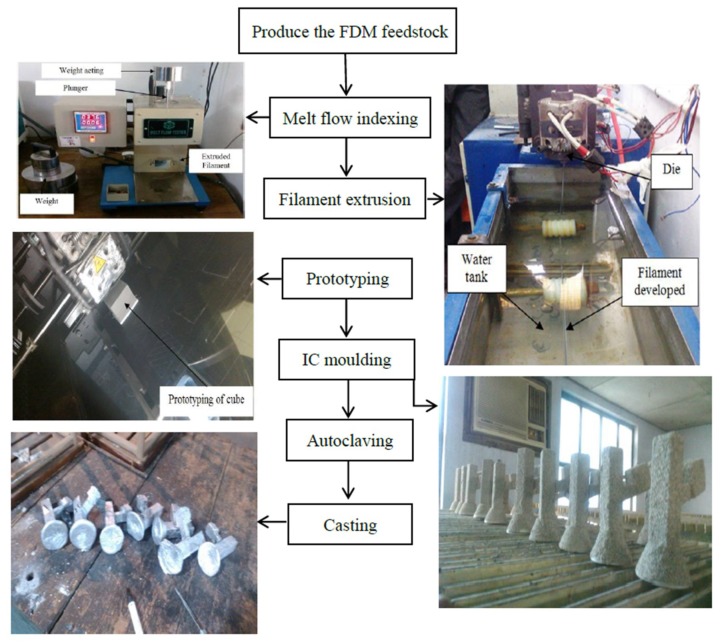
Step by step procedure of aluminium matrix composites (AMC) development.

**Figure 2:**
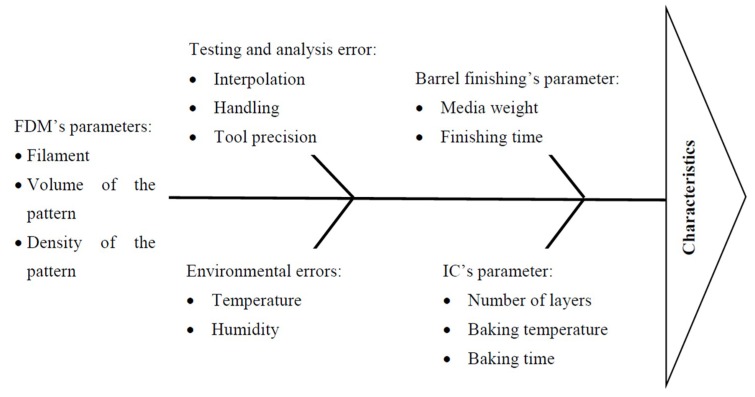
Fish bone diagram for prepared castings.

Figure 1 presents the methodology adopted in this research. By using a Fish bone diagram (see details on Figure 2), we highlight the main process parameters associated to the IC which can affect the quality features of the IC components. The number of IC slurry layer (N_SL_) has been judicially selected as an input parameter due to its significance highlighted in the literature [38,39,40]. The following are the procedural steps followed to obtain AMCs, which refer to the original Taguchi L18 orthogonal array (Table A1), as given in the Appendix A:

- The alternative feedstock filaments (F_P_) have been prepared using PA, Al_2_O_3_, and Al in different %wt. proportions with the help of single screw extrusion process.
- The formed filaments were used for the development of sacrificial patterns of cubical shape with three different volumes (V_P_), such as 17,576 mm^3^, 27,000 mm^3^, and 39,304 mm^3^. They were produced at low, high, and solid density of FDM process (D_P_) by using uPrint-SE system of Stratasys Inc. (Edina, MN, USA). In the works, reported previously, it has been seen that the change in the in-fill density affects the mechanical and tribological performances of the developed AMCs [1,2,3]. The prime reason behind the selection of FDM technology is due to its affordability and suitability for hybridization within the IC process [23,24,41]. Further, the selection of the process parametric levels from previous studies has been judicially selected, based on the pilot studies.
- Prior to shell moulding, the barrel finishing (BF) process was performed on the samples, for the refurbishment of resulted surface finish [31]. Here, barrel finishing time (BF_T_) and barrel finishing media weight (BF_W_) have been selected as input process parameters.
- Then, the IC moulds were prepared by coating the trees (consisting of riser, pouring basin, gating, and also the FDM printed sacrificial pattern) with refractory layers of silica. The number of IC slurry layers (N_SL_) has also varied in accordance to Table A1 in the Appendix A.
- Autoclaving and baking were performed in one step at 1150 °C (by maintaining the pouring sprue in a vertical up position so that the Al_2_O_3_ filler particles could be arrested within the cavity only). At this range of temperature, the matrix of the sacrificial patterns evaporates, immediately, without causing mould cracks.
- Finally, pouring of molten Al-6063 has been carried out.

**Figure 3:**
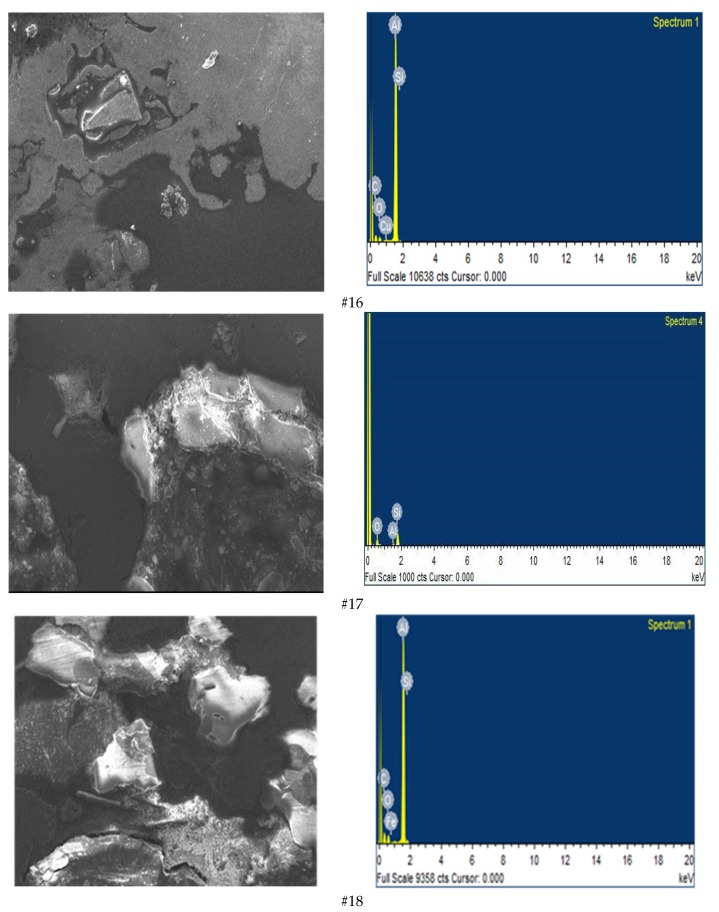
SEM micrograph and EDS spectrum of experiment #16, #17, and #18.

The castings manufactured were tested for surface hardness, dimensional accuracy and surface roughness by using HVS-1000BVM hardness tester (HV0.01 scale; ASTM-E384, Laizhou, China), Vernier Caliper (Mitutoyo: least count 0.01mm, Takatsu-ku, Kawasaki, Japan) and Mitutoyo SJ-210 (Japan, ISO: 1997) surface roughness tester, respectively. For microstructural evaluation, the Scanning Electron Microscopy (SEM, JEOL, Peabody, MA, USA) analysis has been performed on the casting manufactured in the experiment #16, #17 and #18 associated to Table A1. It has been seen that the Al_2_O_3_ particles presented in Al matrix allow to enhance the quality characteristics of the castings, especially the hardness on the surface. Figure 3 shows the SEM micrographs and their associated Energy Dispersive Spectroscopy (EDS) spectrums (JEOL, USA). The measurements indicate the presence of Al, O, Si, Fe, and C-peaks, which confirm the existence of alumina. These elements identified on the EDS measurements (i.e., Al, O, and C) are the common sign of alumina surface [42]. They were noted as well as the presence of elements Fe and Si, which denote some small impurity. 

## 3. Dimensionless modelling: Buckingham Pi Approach

**Figure 4:**
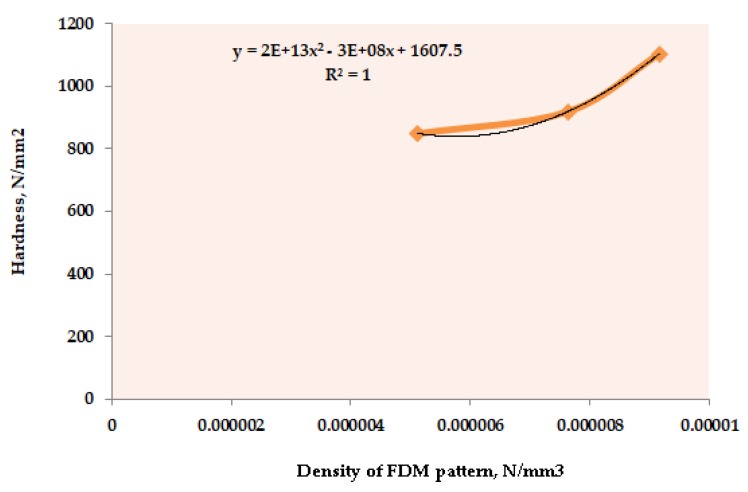
Hardness versus density of fused deposition modelling (FDM) pattern plot.

Dimensionless modelling of the experimental data is considered an efficient method in order to formulate analytic mathematical functions that are out of a highly complex experimental system associated to numerous process parameters [43]. The concept of dimensionless analysis helps to reduce the influence of variables by means of physical equations [44,45,46]. To date, dimensionless modelling with the help of Buckingham Pi approach has been extensively investigated for a wide range of scientific and engineering applications including fluid dynamics [47], energy [48], electronics [49], heat transfer [50] and others. According to the Buckingham approach, any practical problem containing “n” factor sand further “m” dimensions, then the subtraction of n and m will result the counts of independent factors, which could be assumed. Presently, “n” and “m” are 7 and 3, respectively. Therefore, the problem will consist of π1, π2, π3 and π4 that are the dimensional magnitudes. Furthermore, the mathematical formulae derived for the assumed independent parameters help to develop the dimensional relationships by following a set of standard steps [51,52]. Standard quantities of the same physical nature (mass, length, and time) are used based on fundamental units. Consequently, it can be said that these systems belong to the same class. To generalize, a set of systems of units that differ only in the magnitude (but not in the physical nature) of the fundamental units are called a class of systems of units [53]. Unlike other statistical approaches, the mathematical modelling in the case of Buckingham’ Pi approach could be very tedious if a proper set of producers is not considered. Based on [53], following are the step-by-step descriptions of the modelling process adopted in the present work:

- First of all, the units of the input and the output process parameters have been unified and converted into physical quantities (such as M, L, and T). Further, it is of utmost importance to highlight that any kind of categorical parameter, either input or output, is not suitable for the modelling. Moreover, upon such conversions, it should be considered that the replacement could be represented in-terms of M, L, and T formats. Therefore, in present work, the original Table A1 in the Appendix A has been modified in order to balance the units, as well as to convert the qualitative parameters into quantitative. For instance, the parameter “filament proportion” has been quantified in-terms of its tensile strength; density of the FDM pattern has been considered in terms of mass and volume; mould wall thickness has been converted from a number of layers to thickness of the wall, etc. Table 1 is the final prepared modified version of Table A1.
  The obtained dimensions of input and output parameters would be:
  Hardness (H) as ML^−1^T^−2^,
  Dimensional accuracy as L,
  Surface roughness as L,
  Filament proportion (P) in-terms of tensile strength of filament as MLT^−2^,
  Volume of FDM reinforced pattern (V) as L^3^,
  Density of FDM pattern (ρ) as ML^−3^,
  BF cycle time (t) as T,
  BF media weight (W) as M and the Number of IC slurry layers (l) resulting into mould wall thickness as L.
- Then, it is mandatory to find out the significance level of the input process parameters for the measured outcomes. In the present case, ANOVA has been implemented with the help of MINITAB-17 based statistical software in order to identify the significance and contribution of input parameters. Table 2 shows the contribution percentage of input process parameters for surface hardness, dimensional accuracy, and surface roughness.
- Before starting to formulate the π equations (let us say ‘x’), it is necessary to identify the ‘x − 1’ top performing input parameters. For instance, in the case of surface hardness, when ‘x’ is equal to 4 that allows to develop 4 π-equations, three top performing input parameters have to be identified.
- Now, the top performing input parameters and the output parameters being analyzed represent the π equations.
- After calculating the π equations, the π1 (related to the output parameter) is solved as a function of other πs (π2, π3, and π4, consisted of input parameters).
- Once the step-v is completed, a constant ‘K’ has been considered whose value has been driven from a second order quadratic equation of the fitness curve that connect the output response and the most contributing input parameter.
- Further, the fitness curve should be plotted between the measured output values and the corresponding values of the most significant input parameter, while keeping the rest of the parameters constant. Alternatively, in the present case, the plots have been drawn between the three levels of the input process parameters and the average of the corresponding output result. For instance, in case of Figure 4, the average of hardness for experiment #1, #4, #7, #10, #13, and #16 has been plotted against first level of F_D_ (5.12 × 10^−6^ N/mm^3^) and the average of hardness for experiment #2, #5, #8, #11, #14, and #17 has been plotted against second level of F_D_ (7.63 × 10^−6^ N/mm^3^). Similar procedure has been adopted for the third level of the F_D_.
- Noticeably, the regression (R^2^) ~ 1 indicates the best fitness of the data.

### 3.1. Hardness

In the present study, hardness is considered a function of all input process parameters that is expressed by Equation (1). 

So,

H = f(P, V, ρ, t, W, l)(1)

Based on the Table 2; the least significant parameters for this particular parameter are BF cycle time, BF media filament proportion, and weight that will directly go in “π” groups. The “π” eqns. for hardness can be written as:

π_1_ = H (F)^a1^ (t)^b1^ (W) ^c1^ (2)

π_2_ = ρ (F)^a2^ (t)^b2^ (W)^c2^ (3)

π_3_= l (F)^a3^ (t)^b3^ (W)^c3^ (4)

π_4_ = V (F)^a4^ (t)^b4^ (W)^c4^ (5)

After substituting the decided dimensions in the “π” groups, Equations (6), (8), (10), and (12) are formed. Now, in order to solve these further, the resulted equations are equated to zero. For instance, the π1 will be solved as follows:

π_1_ = ML^−1^T^−2^ (ML^−1^T^−2^)^a1^ (T)^b1^ (M) ^c1^ (6)

Equating the basic dimensions to zero:

M: 1 + a_1_ + c_1_ = 0 

L: −1 − a_1_ = 0 

T: −2 −2a_1_ + b_1_ = 0 

We get,

a_1_ = −1, b_1_ = 0 and c_1_ = 0

So, Equation (2) can be re-written as:

π_1_ = H/F (7)

Similarly, on solving π_2_;

π_2_ = ML^−3^ (ML^−1^T^−2^)^a2^ (T)^b2^ (M)^c2^ (8)

Similarly, equating the basic dimensions to zero:

M: 1 + a_2_ + c_2_ = 0 

L: −3 − a_2_ = 0 

T: −2a_2_ + b_2_ = 0 

We get, 

a_2_ = −3, b_2_ = −6 and c_2_ = 2

So, Equation (3) can be re-written as;

π_2_ = ρ/F^3^t^6^ (9)

On solving π_3_;

π_3_ = L (ML^−1^T^−2^)^a3^ (T)^b3^ (M^)c3^ (10)

Equating the basic dimensions to zero:

M: a_3_ + b_3_ = 0 

L: 1 − a_3_= 0 

T: −2a_3_ + b_3_ = 0 

We get, 

a_3_ = 1, b_3_ = 2 and c_3_ = −1

The Equation (4) for π_3_ can be re-written as;

π_3_ = lFT^2^/W (11)

Solving π_4_;

π_4_ = L^3^ (ML^−1^T^−2^)^a4^ (T)^b4^ (M)^c4^ (12)

Equating the basic dimensions to zero:

M: a_4_ + c_4_ = 0 

L: 3 – a_4_ = 0 

T: 0 – 2a_4_ + b_4_ = 0 

We get, 

a_4_ = 3, b_4_ = 6 and c_4_ = −3

The Equation (5) for π_4_ can be re-written as;

π_4_ = VF^3^t^6^/W^3^ (13)

The final relationship between all four Equations of “π” can be assumed as;

π_1_ = f(π_2_,π_3_ and π_4_)

Or

H/F =$\left(\frac{\rho }{F^{3}t^{6}},\frac{{\mathrm{lFt}}^{2}}{W}{\mathrm{and}\;\mathrm{VF}}^{3}t^{6}/W^{3}\right)$

The above expression can be written as:

H = K·ρ·F^2^·l·t^2^·V/W^4^ (14)

Here, “K” is the proportionality constant.

Experimentally, it has been found that a correlation between the hardness and “ρ” exists (refer Table 2). Hence, it was taken as representative factors to develop the mathematical model. The average values of the hardness obtained at different levels of “ρ” (throughout the Table 1) has been plotted (see details in Figure 4). In this case, a regression equation (R^2^ = 1) with a second order has been determined. Based on the obtained linear equation, the final mathematical model that includes the hardness is given:

H = [(2E + 13ρ ^2^ – 3E + 8ρ + 1607.5)]F^2^·L·t^2^·V/w^4^ (15)

### 3.2. Dimensional Accuracy

In a similar way, dimensional accuracy is considered as a function of all input process parameters that is expressed by Equation (16). 

(16) Δd =f(F, V, ρ, t, W, l)

From Table 2, the least significant parameters are BF cycle time, number of IC slurry layers, and filament proportion, that will directly go in “π” groups. The “π” equation for dimensional accuracy can be written as:

π_1_ = Δd (F) ^a1^ (t) ^b1^ (L) ^c1^ (17)

π_2_ = W (F) ^a2^ (t) ^b2^ (L) ^c2^ (18)

π_3_ = ρ (F) ^a3^ (t) ^b3^ (L)^c3^ (19)

π_4_ = V (F) ^a4^ (t) ^b4^ (L) ^c4^ (20)

The same set of mathematical iterations has been repeated for dimensional accuracy and the relationship between the all four “π” equations is given in Equation (21) as below:

(21) $$\Delta d/l\;=\;f\left(\frac{W}{Ft^{2}l},\frac{{\rho l}^{2}}{{\mathrm{Ft}}^{2}}\mathrm{and}\frac{V}{l^{3}}\right)$$

On solving the above expression, we get:

Δd = K·ρ·W·V/F^2^·l·t^4^ (22)

**Figure 5:**
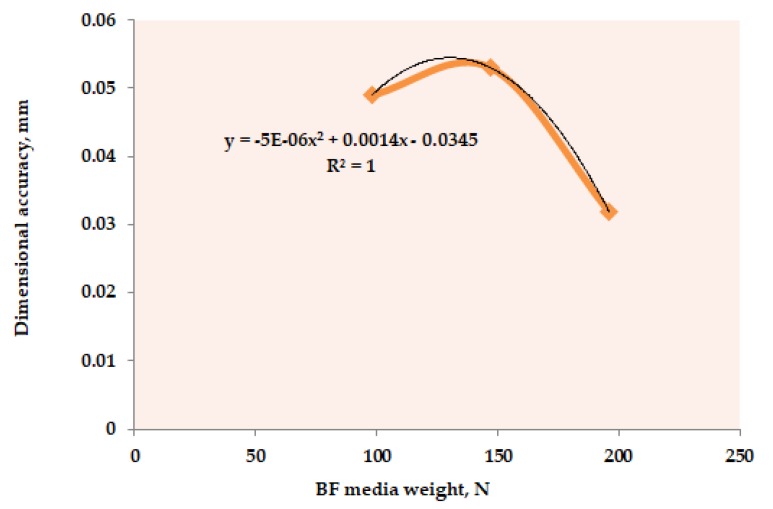
Dimensional accuracy versus barrel finishing (BF) media weight plot.

BF media weight, which is the most significant parameter (refer to Table 2) with regards to dimensional accuracy, of the casted composites, has been taken as the representative parameter to develop the mathematical model. For this, the average values of the dimensional accuracy obtained at different levels of “BF_W_” (throughout the Table 1) has been plotted; refer to Figure 5. Then, a regression equation (R^2^ = 1) with a second order has been determined. Based on the obtained linear equation, the final mathematical model for dimensional accuracy is given as:

Δd = [(−5E − 06W^2^ + 0.0014W – 0.0345] ρ·V/F^2^·l·t^4^ (23)

### 3.3. Surface Roughness

Further, Equation (24) represents the surface roughness, as a function of all input process variable:

(24) Ra =f(F, V, ρ, t, W, l)

Based on the Table 2; density of FDM pattern, filament proportion, and BF media weight are the least significant parameters for surface roughness that will directly go in “π” groups. The “π” equation for dimensional accuracy can be written as:

π_1_ = Ra (W) ^a1^ (ρ) ^b1^ (F) ^c1^ (25)

π_2_ = V (W) ^a2^ (ρ) ^b2^ (F) ^c2^ (26)

π_3_ = t (W) ^a3^ (ρ) ^b3^ (F) ^c3^ (27)

π_4_ = l (W) ^a4^ (ρ) ^b4^ (F) ^c4^ (28)

Now, repeating the same set of mathematical operations the final expression that describes the relationship between all the four “π” is given as Equation (29):

(29) $$R_{a}{\left(\rho /W\right)}^{1/3}=\;f\left(\frac{V\rho }{W},\frac{t}{{\left(\rho \right)}^{\frac{1}{6}}{\left(\mathrm{FW}\right)}^{\frac{1}{3}}}\mathrm{and}\;l{\left(\frac{W}{\rho }\right)}^{1/3}\right)$$

Equation (29) can be written as:

Ra = K·(V·t·l·ρ^^(1/6)^)/F^1/3^·W^2/3^, (30)

**Figure 6:**
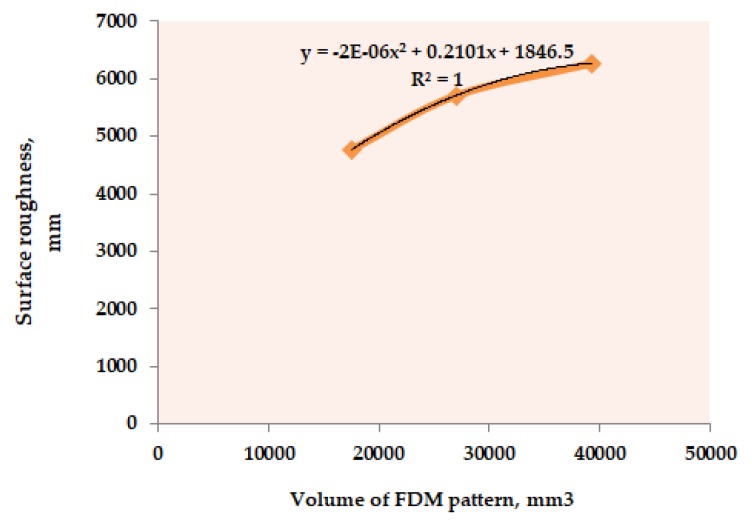
Surface roughness versus volume of FDM pattern plot.

Similar to the dimensional accuracy, volume of FDM reinforced pattern which is the most significant parameter (refer to Table 2) with regard to surface roughness, of the casted composites, has been taken as the representative parameter to develop the mathematical model. For this, the average values of the surface roughness obtained at different levels of “V_P_” (throughout Table 1) has been plotted; refer to Figure 6. Then, a regression equation (R^2^ = 1) with a second order has been determined. From the obtained linear equation, the final mathematical model for surface roughness is given as:

(31) $$R_{a}\;=\;[(-2E\;-\;6V^{2}\;+\;0.2101V\;+1846.5)]\cdot (t\cdot l\cdot \rho^{\frac{1}{6}})/F^{1/3}\cdot W^{2/3}$$

These results obtained in the present work are found to be in-line with the observations presented in the literature [42,45].

## 4. Conclusions

In this work, Vashy-Buckingham’s π-theorem was employed successfully for the development of the mathematical models related to the hardness, dimensional accuracy, and surface roughness of AMCs; material that was produced through FDM assisted by the IC process. The ANOVA simulation were embedded in the present methodology in order to generate a standard database and to recognize the significance process parameters, respectively. Further, all three mathematical models developed are of second order polynomial equations, with a regression value equal to 1, which prove the reliability of the models. 

## Figures and Tables

**Table 1:** Modified Taguchi L18 orthogonal array.

Exp. No.	Tensile Strength, N/mm^2^	Volume of Fused Deposition Modelling (FDM) Reinforced Pattern (mm^3^)	Density of FDM Pattern, N/mm^3^	BF Cycle Time (sec)	BF Media Weight (N)	Mould Wall Thickness Obtained, mm	H, N/mm^2^ (Converted from HV with a Multiplying Factor of 9.807)	Δd, mm	R_a_, mm (Converted from µm with a Dividing Factor of 0.001)
1	21.65	17576	5.12 × 10^-6^	1200	98	11.5	877.72	0.026	4762
2	21.65	17576	7.63 × 10^−6^	2400	147	13	900.28	0.033	5151
3	21.65	17576	9.16 × 10^−6^	3600	196	15	1127.80	0.02	4778
4	21.65	27000	5.12 × 10^−6^	1200	147	13	787.50	0.056	4371
5	21.65	27000	7.63 × 10^−6^	2400	196	15	848.30	0.063	5582
6	21.65	27000	9.16 × 10^−6^	3600	98	11.5	1127.80	0.053	6094
7	21.65	39304	5.12 × 10^−6^	2400	98	15	756.11	0.043	5368
8	21.65	39304	7.63 × 10^−6^	3600	147	11.5	901.26	0.08	5658
9	21.65	39304	9.16 × 10^−6^	1200	196	13	984.62	0.016	6404
10	21.53	17576	5.12 × 10^−6^	3600	196	13	915.97	0.016	4709
11	21.53	17576	7.63 × 10^−6^	1200	98	15	940.49	0.076	4573
12	21.53	17576	9.16 × 10^−6^	2400	147	11.5	1317.08	0.056	4658
13	21.53	27000	5.12 × 10^−6^	2400	196	11.5	934.60	0.033	5297
14	21.53	27000	7.63 × 10^−6^	3600	98	13	919.89	0.05	5889
15	21.53	27000	9.16 × 10^−6^	1200	147	15	1024.83	0.06	6845
16	21.53	39304	5.12 × 10^−6^	3600	147	15	824.76	0.033	8564
17	21.53	39304	7.63 × 10^−6^	1200	196	11.5	1004.23	0.043	5721
18	21.53	39304	9.16 × 10^−6^	2400	98	13	1041.50	0.046	5894

**Table 2:** Percentage contribution of input process parameters.

Source	Surface Hardness (H)	Dimensional Accuracy (Δd)	Surface Roughness (Ra)
F_P_	7.69%	0.76%	4.16%
V_P_	8.85%	16.95%	43.84% *
D_P_	65.75% *	19.83%	3.03%
BF_T_	1.03%	3.30%	6.45%
BF_W_	0.8 %	31.71% *	2.94%
N_SL_	14.14%	8.97%	5.72%
Residual Error	1.74%	18%	33.86%
Total	100%	100%	100%

* Highly contributing factor.

## Appendix A

**Table A1:** Design of experimentation as per original Taguchi L18 orthogonal array.

Exp. No.	F_P_	V_P_ (mm^3^)	D_P_	BF_T_	BF_W_	NS_L_	R_a_ (µm)	S/N ratio (dB)	Δd (mm)	S/N ratio (dB)	HV	S/N ratio (dB)
1	C1	26 × 26 × 26	Low density	20	10	7	4.762	−13.55	0.026	31.34	89.5	39.01
2	C1	26 × 26 × 26	High density	40	15	8	5.151	−14.27	0.03	29.45	91.8	39.18
3	C1	26 × 26 × 26	Solid	60	20	9	4.778	−13.58	0.02	33.30	115	41.18
4	C1	30 × 30 × 30	Low density	20	15	8	4.371	−12.82	0.06	23.37	80.3	38.06
5	C1	30 × 30 × 30	High density	40	20	9	5.582	−14.93	0.063	23.80	86.5	38.72
6	C1	30 × 30 × 30	Solid	60	10	7	6.094	−15.69	0.053	25.32	115	41.22
7	C1	34 × 34 × 34	Low density	40	10	9	5.368	−14.59	0.043	27.06	77.1	37.73
8	C1	34 × 34 × 34	High density	60	15	7	5.658	−15.05	0.08	21.89	91.9	39.25
9	C1	34 × 34 × 34	Solid	20	20	8	6.404	−16.13	0.016	35.22	100.4	39.92
10	C2	26 × 26 × 26	Low density	60	20	8	4.709	−13.45	0.016	35.22	93.4	39.38
11	C2	26 × 26 × 26	High density	20	10	9	4.573	−13.20	0.076	22.29	95.9	39.62
12	C2	26 × 26 × 26	Solid	40	15	7	4.658	−13.36	0.056	24.72	134.3	42.60
13	C2	30 × 30 × 30	Low density	40	20	7	5.297	−14.48	0.033	29.45	95.3	39.56
14	C2	30 × 30 × 30	High density	60	10	8	5.889	−15.40	0.050	25.90	93.8	39.41
15	C2	30 × 30 × 30	Solid	20	15	9	6.845	−16.70	0.060	24.35	104.5	40.37
16	C2	34 × 34 × 34	Low density	60	15	9	8.564	−18.65	0.033	29.20	84.1	38.29
17	C2	34 × 34 × 34	High density	20	20	7	5.721	−15.15	0.043	27.06	102.4	40.20
18	C2	34 × 34 × 34	Solid	40	10	8	5.894	−15.40	0.046	26.44	106.2	40.48

Where, F_P_, V_P_, D_P_, BF_T_, BF_W_, NS_L_, Ra, Δd, HV, and S/N represent the filament proportion, volume of the pattern, density of the pattern, barrel finishing time, barrel finishing media weight, number of IC slurry layers, surface roughness, dimensional accuracy/deviation, Vickers hardness, signal/noise, respectively. Further, C1 and C2 are the compositions of PA_x_/Al_2_O_3y_/Al_z_ (where x is 60% by wt.; y is 10% and 12% by wt., respectively; and z: 28% and 30% by wt., respectively).
